# Quantification of the effect of in utero events on lifetime resilience in dairy cows

**DOI:** 10.3168/jds.2023-24215

**Published:** 2024-07

**Authors:** Katharine Lewis, Laura Shewbridge Carter, Andrew Bradley, Richard Dewhurst, Niamh Forde, Robert Hyde, Jasmeet Kaler, Margaret D. March, Colin Mason, Luke O'Grady, Sam Strain, Jake Thompson, Martin Green

**Affiliations:** 1 Department of Veterinary Medicine and Science, University of Nottingham, Nottingham LE12 5RD, United Kingdom; 2 Scotland's Rural College, Edinburgh AB21 9YA, United Kingdom; 3 Quality Milk Management Services, Cedar Barn, Easton, Wells, United Kingdom; 4 Discovery and Translational Sciences Department, Leeds Institute of Cardiovascular and Metabolic Medicine, School of Medicine, University of Leeds, Leeds BA5 1DU, United Kingdom; 5 Animal Health and Welfare Northern Ireland, Dungannon, Co. Tyrone, BT71 6JT, United Kingdom

**Keywords:** dairy cow resilience, developmental origins of health and disease, heat stress

## Abstract

Currently, the dairy industry is facing many challenges that could affect its sustainability, including climate change and public perception of the industry. As a result, interest is increasing in the concept of identifying resilient animals, those with a long productive lifespan, as well as good reproductive performance and milk yield. There is much evidence that events in utero, that is, the developmental origins of health and disease hypothesis, alter the life-course health of offspring and we hypothesized that these could alter resilience in calves, where resilience is identified using lifetime data. The aim of this study was to quantify lifetime resilience scores (LRS) using an existing scoring system, based on longevity with secondary corrections for age at first calving and calving interval, and to quantify the effects of in utero events on the LRS using 2 datasets. The first was a large dataset of cattle on 83 farms in Great Britain born from 2006 to 2015 and the second was a smaller, more granular dataset of cattle born between 2003 and 2015 in the Langhill research herd at Scotland's Rural College. Events during dam's pregnancy included health events (lameness, mastitis, use of an antibiotic or anti-inflammatory medication), the effect of heat stress as measured by temperature-humidity index, and perturbations in milk yield and quality (somatic cell count, percentage fat, percentage protein and fat:protein ratio). Daughters born to dams that experienced higher temperature-humidity indexes while they were in utero during the first and third trimesters of pregnancy had lower LRS. Daughter LRS were also lower where milk yields or median fat percentages in the first trimester were low, and when milk yields were high in the third trimester. Dam LRS was positively associated with LRS of their offspring; however, as parity of the dam increased, LRS of their calves decreased. Similarly, in the Langhill herd, dams of a higher parity produced calves with lower LRS. Additionally, dams that recorded a high maximum locomotion score in the third trimester of pregnancy were negatively associated with lower calf LRS in the Langhill herd. Our results suggest that events that occur during pregnancy have lifelong consequences for the calf's lifetime performance. However, experience of higher temperature-humidity indexes, higher dam LRS, and mothers in higher parities explained a relatively small proportion of variation in offspring LRS, which suggests that other factors play a substantial role in determining calf LRS. Although “big data” can contain a considerable amount of noise, similar findings between the 2 datasets indicate it is likely these findings are real.

## Abstract (author highlights)

The list of standard abbreviations for JDS is available at adsa.org/jds-abbreviations-24. Nonstandard abbreviations are available in the Notes.

## INTRODUCTION

In light of the current challenges facing the dairy industry, such as climate change and public perception, interest is growing in the concept of cow resilience as a way to increase the sustainability of dairy farming. Resilience encompasses an animal's capacity to cope with environmental, social, and disease challenges, and cows that are considered resilient have a high probability of completing many lactations with a good reproductive performance and few health problems (Ahlman et al., 2011; Adriaens et al., 2020; Ouweltjes et al., 2021). Resilient cows therefore cope well with the farm's management and environmental conditions and avoid premature culling.

One factor that influences an individual's lifetime health (and by proxy their resilience) is the in utero environment in which they were gestated—known as developmental origins of health and disease. Substantial evidence exists for this phenomenon in humans; for example, several epigenetic effects are associated with prenatal exposure to hunger (Vaiserman and Lushchak, 2021). It has already been demonstrated in cattle that nutritional restriction can alter numbers of oocytes in an animal's ovarian reserve (Mossa et al., 2009) and in utero heat-stressed heifers have mammary glands with smaller alveoli (Skibiel et al., 2018a). Being able to identify resilient animals based on the events experienced by their mothers during pregnancy could help inform farm management decisions. In particular, the effect of heat stress on fetus development is of interest to the dairy industry because climatic disturbances are likely to increase as a result of climate change (Hansen et al., 2012). When the effect of heat stress on the fetus is known, the importance of preventing heat stress is better substantiated.

To quantify resilience, indicative traits are used because resilience itself is difficult to measure directly. A definition of resilience agreed on by the EU Horizon 2020 GenTORE consortium (Friggens and De Haas, 2019) is that resilience can be considered as the cumulative effect of good health and fertility, resulting in a long productive life span. Using this definition, quantitative lifetime resilience scores (**LRS**) can be calculated by allocating points based primarily on the number of lactations completed and the cow's productive performance relative to the rest of the herd (Adriaens et al., 2020; Ouweltjes et al., 2021). These scores allow resilience to be identified from commercially available data, but do not account for factors that may vary within farms, such as changes in management over time (Adriaens et al., 2020). Although it is possible to quantify resilience using these scores, there is limited knowledge about factors associated with between-cow heterogeneity in resilience score.

Developmental origins of health and disease (Barker, 2007; Fleming et al., 2015) suggests that events experienced in very early life, from the periconception period to birth, have lifelong effects. In dairy cows, these environmental sources of stressors include disease events, metabolic and nutritional status, or environmental disturbances, such as high environmental temperature or humidity. Evidence exists that disease experienced by mothers during pregnancy is associated with performance of the offspring; daughters born to mothers that had experienced clinical health events around conception had fewer incidences of disease themselves as young heifers or first-lactation animals (Carvalho et al., 2020) and those from dams with higher mean somatic cell counts had a greater age at first calving, increased first- and second-lactation mean SCC, and reduced yield (Swartz et al., 2021). These changes may occur because the inflammatory response of the dam results in postnatal adaptations in the calf, which induce adaptive changes in the conceptus that may improve its tolerance to postnatal health problems. This has been demonstrated in mouse models where adult offspring of mothers that experienced immune challenge while pregnant are hypersensitive to inflammatory stimuli (Williams et al., 2011). The exact mechanism for this in cattle is currently unknown, but possible pathways include a suboptimal uterine environment (Aiken and Ozanne, 2014), inheritance of mitochondrial dysfunction (Igosheva et al., 2010), or epigenetic alterations (Ozanne and Constância, 2007).

In the UK, cattle currently experience relatively few days of heat stress (Dunn et al., 2014) but by the end of the 21st century, heat-stress events are likely to increase (Fodor et al., 2018). Heat stress experienced during gestation has been found to have detrimental effects; calves born to mothers that experienced heat stress in late gestation have lower birth and weaning weights (Collier et al., 1982; Tao et al., 2012), as well as lower probability of survival and reduced lifetime performance (Monteiro et al., 2016; Weller et al., 2021). Some possible reasons for this could either be that heat stress alters maternal physiology, resulting in increased maternal core body temperature and changes in placental mass and blood flow, which leads to dysfunction (Reynolds et al., 1990, 2006; Van Eetvelde et al., 2016), or heat stress alters maternal behavior, for example heat-stressed mothers reduce their feed intake and alter their lying behavior (Mallonée et al., 1985; Allen et al., 2015; Kanjanapruthipong et al., 2015). These alterations in behavior can then lead to physiological changes; for example, when heat-stressed animals take in less dry matter, protein reserves are mobilized to prioritize the fetus (Lamp et al., 2015). Effects of heat stress can persist long after the developmental insult occurs; exposure to heat stress while in utero results in alterations in mammary gland gene expression (Skibiel et al., 2018a), and these cows produce less milk as heifers (Monteiro et al., 2016).

The purpose of this research was to identify cow- and farm-level maternal stressors that may modify lifetime resilience in the offspring of dairy cows. Specifically, we aimed to investigate the effects of a variety of stressors experienced by the mother during specific stages of pregnancy on individual cow LRS in 2 datasets, one large dataset consisting of cows born over a 10-yr period from 83 farms and a smaller, more granular dataset from 293 animals in the Langhill research herd at Scotland's Rural College over a 12-yr period. These environmental stressors included health-related stress in the dam (mastitis, lameness, and diseases requiring use of antibiotics or anti-inflammatories) and broader environmental stresses associated with heat-stress events defined from national weather stations.

## MATERIALS AND METHODS

### Data Sources

Two different data sources were used. Because no live animal subjects were used, this analysis did not require approval by an Institutional Animal Care and Use Committee. The first was a large dataset that consisted of multiple herds with commercially recorded data (described below). “Big data” has many advantages for creating meaningful insights into animal health (VanderWaal et al., 2017), but farmers differ in their observations of animal health and event recording. In particular, recording of treatments is often lower than the true on-farm use (Nobrega et al., 2017), but the ease of recording and storing data on the farm and the requirements for doing so have increased over time. Therefore, to further investigate our hypothesis, we also considered data from a research herd, where events were recorded with a high level of accuracy and consistency. The 2 datasets are described below:

#### Dataset 1

Herds came from a convenience sample of 108 herds that supplied data to Quality Milk Management Services (Wells, UK). Data were extracted from *TotalVet*, a dairy herd analysis software (https://www.total-vet.co.uk/), into.csv files. The files contained 12,309,843 records from 108 farms dated from July 15, 1975, to June 9, 2022. Records included in the dataset included calving events, milk recordings, and health and treatment events.

#### Dataset 2

This dataset came from cows in the Langhill research herd, housed at the Crichton Royal Farm at the Dairy Research and Innovation Centre at Scotland's Rural College. Data were extracted from a Microsoft SQL Server for cows in the herd born between January 1, 2003, and December 31, 2015, giving records up to the year of data analysis (2022).

### Data Processing and Sample Selection

#### Dataset 1: Selection of Animals

Cows were selected that were born between January 1, 2006 and December 31, 2015 to ensure lifetime data were available for each animal. Data cleaning took place in Python v3.10.5 using *pandas* (McKinney, 2010) and *numpy* (Harris et al., 2020); a summary of the data cleaning steps is detailed in Table 1. In brief, cows were excluded when identification numbers were duplicated, ages at first calving were unrealistic (<15 mo or >4.5 yr), or they were not born on the farm where data were recorded (Table 1). Milk records were selected for each lactation (Table 1) and cows were excluded if milk records occurred before their first recorded calving date, indicating they were not first-parity cows and therefore not all lifetime data were available. Milk records were excluded if the yield was unrealistic (>100 kg/day). The 305-d milk yield for each lactation was calculated using the *milkbot* model, a nonlinear lactation model that uses 4 parameters to fit curves to the lactation (Ehrlich, 2011). Lifetime resilience scores were then calculated (see the “Calculation of LRS” section) for all cows that had calved at least once on the farm.

**Table 1:** Selection of cows and herds for inclusion in the final models of lifetime resilience score for dataset 1

Selection step^1^	Number of animals	Number of records	Number of herds
Animal records	338,129	—	108
Cow identification number occurred on one farm only	336,423	—	
Cows entered herd on their date of birth	309,065	—	
Cow had not had a previous lactation on entry to herd	218,929	—	
Cows born 2006–2015	84,795	—	
Calving records	56,500	206,362	
Age at first calving >458 d and <1,461 d	56,009	204,539	
Milk records	54,940	2,193,071	
Milk records selected between calving date*_j_* and calving date*_j_*_+ 1_, or after the last calving date*_jn_*	53,849	2,148,907	
Records with yield >100 kg removed	53,358	2,148,837	
*milkbot* model applied to records DIM ≥0 and ≤305 d and yield >0	52,030	1,800,013	
Cows excluded if yield was 0 in any lactation but lactation *j*	45,425	159,744	
LRS calculated	45,317	—	102
>1 stressor recorded in the year by the farm	43,500	149,351	101
LRS for years where there was recording and mother–daughter pairs could be matched	42,982	—	83
Mother–daughter pairs matched in recording years	15,838 daughters 12,125 mothers	—	83
First calves excluded	9,292 daughters 7,334 mothers	—	83

1 Lactation *j* = the lactation starting from the last recorded calving date of the cow. Cows were excluded if the yield was 0 in any lactation but *j*. LRS = lifetime resilience score. LRS was not calculated for 127 animals that were first parity, with no milk data for the lactation.

Because of variability in recording of treatments between herds and years within herds, herd-years were only included in the analysis when at least one “stressor” (lameness, mastitis, or treatment with antimicrobial or anti-inflammatory products) was recorded in the year. Once mother–daughter pairs had been matched up, the dataset consisted of 15,838 mother–daughter pairings, where the daughter had calved at least once and therefore had her own LRS. The first calves from each cow were excluded because the mother was not lactating during that pregnancy meaning the effect of production-related variables could not be assessed.

#### Dataset 2, Langhill Research Herd: Selection of Animals

Cows were selected that were born between January 1, 2003, and December 31, 2015, to ensure lifetime data were available for each animal. The Langhill research herd contains 2 genetic lines: a control genetic group (UK average production efficiency) and a select group (high production efficiency; Pollott and Coffey, 2008). The herd continuously hosts feed-trial research, which occurs in 5-yr cycles. During this research period, feed trials had cows grouped in either high-input, all-year-round housed systems or low-input, seasonal grazing systems and once assigned to a system, cows did not change system as feed trials changed.

Due to the smaller size of the initial dataset, data were systematically assessed in Microsoft Excel. Criteria for selection were that cow service dates corresponded to the relevant calving date and that all milk recording data were available. Cows without these data were removed from the dataset. The final dataset consisted of 192 mother–daughter pairings and 74 mother–granddaughter pairings (Table 2).

**Table 2:** Selection of cows for inclusion in the final models of lifetime resilience score for dataset 2 (Langhill herd)

Selection step	Number of animals
Cows born 2003–2015	928
LRS calculated^1^	811
Mother–daughter pairs matched	390 daughters 293 mothers
Mother–daughter pairs with complete stressor data^2^	192 daughters 156 mothers
Mother–granddaughter pairs matched	158 granddaughters 105 mothers
Mother–granddaughter pairs with complete stressor data^2^	74 granddaughters 53 mothers

1 LRS was not calculated for animals that had incomplete data.

2 Body condition and locomotion scores were not available for first-parity births.

#### Calculation of LRS

An LRS was calculated for cows as in Adriaens et al. (2020), where resilience was based on the cumulative result of the cow's ability to recalve (thereby extending her reproductive lifespan), with secondary corrections applied for age at first calving, 305-d milk yield, and calving intervals. The score consists of a baseline interval equal to the calving interval of the herd and each newly started lactation gains a bonus of 300 points. Each cow then gains or loses points for the following components:

1. For every day shorter or longer that their date of first calving was from 730 d,
2. For the number of days that the calving interval is shorter or longer than the herd average,
3. For the percentage that the 305-d milk yield is higher or lower than the herd average, and
4. Points are lost if the cow exits the herd before 100 DIM.

The LRS was calculated as described by Adriaens et al. (2020):

$$\begin{array}{l} LRS_{i}=\;\bar{CInt}+300\;x\;L_{i}+\left(730-AFC_{i}\right)\\ +\;\sum_{j=1}^{L_{i}-1}\left[\left(\bar{CInt_{j}}-\;\bar{CInt_{i,j}}\right)+\sum_{j=1}^{L_{i}}[\frac{\sum_{k=1}^{\max\left(305,DIM_{i,j}\right)}MY_{i,j,k}}{\sum_{k=1}^{\max\left(305,DIM_{i,j}\right)}\bar{MY_{i,j,k}}}-1\right]\\ \times 100+\min\left[0,\left(DIM_{i,L_{i}}-100\right)\right], \end{array}$$

where *LRS_i_* = lifetime resilience score for cow *i*, $\bar{CInt}$ = average calving interval of the herd over all selected years, *L_i_* = lactation number in which cow *i* exited the herd (last lactation number of a cow), *AFC_i_* = age at first calving of cow *i* (in days), *CInt_i_*_,_*_j_* = calving interval of cow *i* between the start of lactation *j* and (*j* + 1), $\bar{CInt_{i,j}}$ = average calving interval between the start of lactation *j* and (*j* + 1) of all cows in the herd, *MY_i_*_,_*_j_*_,_*_k_* = milk production (in kilograms) of cow *i* at day *k* of lactation *j*, $\bar{MY_{i,j,k}}\;$= average milk production (in kilograms) at day *k* of all cows in the herd in lactation *j*, *DIM_i_*_,_*_j_* = DIM of cow *i* at the end of lactation *j*, *DIM_i_*_,_*_Li_* = DIM of cow *i* at the end of her last lactation *L_i_*.

#### Explanatory Variables

Potential stressor events that could be identified in both datasets came from records of lameness, mobility scores, and treatments given. Climate data were obtained from the National Center for Environmental Information National Oceanic and Atmospheric Administration's Global Summary of the Day (NOAA National Centers for Environmental Information, 1999). The explanatory variables used in both analyses are detailed below.

#### Dataset 1: Health Events, Treatment Records and Milk Quality Records

Health records included for dataset 1 were as follows:

1. Clinical mastitis: the date the cow was recorded with a clinical case of mastitis.
2. Clinical lameness: the date the cow was recorded with a clinical case of lameness.
3. Mobility scores: the date when the cow was identified lame during a routine herd mobility score. These were combined with the clinical lameness records to give records of any identified case of lameness.
4. Treatment or other records: these were recorded as free text, along with the date. A list of products registered as authorized on the Veterinary Medicines Directorate Product Information Database (https://www.vmd.defra.gov.uk/productinformationdatabase/current) was downloaded, and filtered for whether the use category was marked as antimicrobial, anti-inflammatory (or both), or a vaccine. Records were matched to the products using partial ratio string joining with *fuzzywuzzy* (SeatGeek Inc., 2014). This method matches strings by calculating the ratio similarity measure (Levenshtein distance) between strings *x* and *y*. Where the shorter string (*x*) is of length *m*, the measure is calculated between the shorter string and every substring of length *m* of the longer string, and the maximum of those similarity measures is returned. Records were manually checked following joining, and any incorrect matches were removed.
5. Milk quality records included percentage fat, protein, lactose, and SCC at each recording.

#### Farm Location, Climate Records, and Calculation of a Thermal Discomfort Index

**Figure 1:**
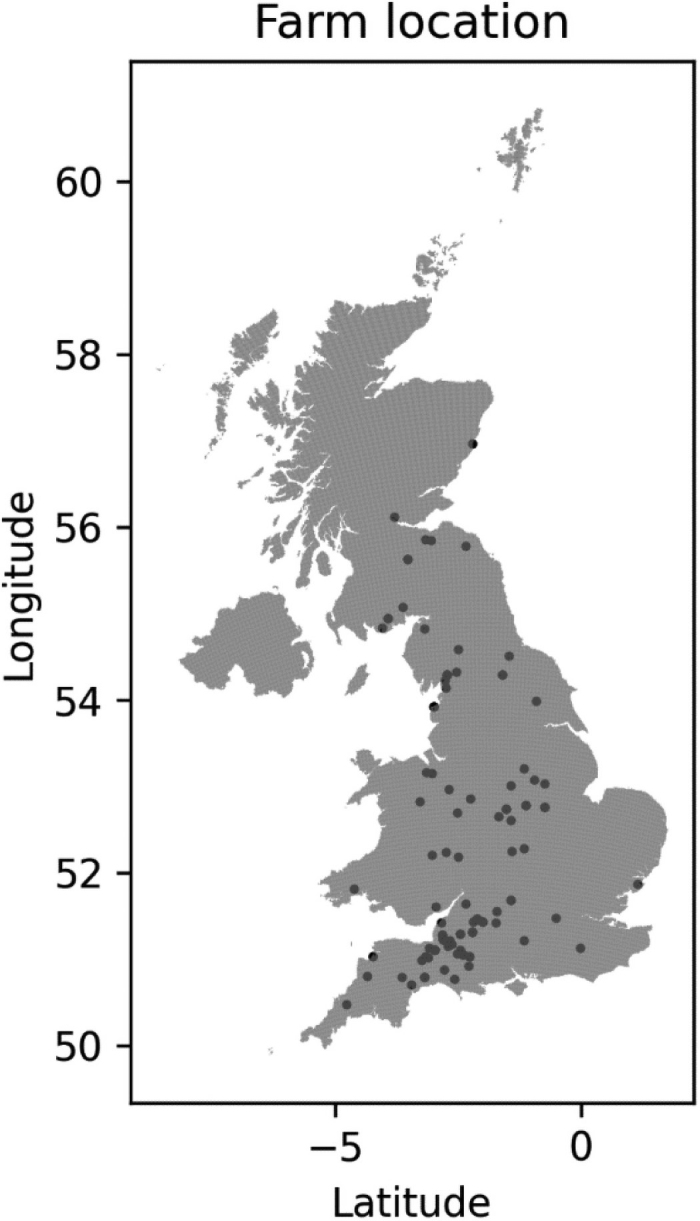
Point locations of the “outcode,” which is the first 4 letters of the postcode, corresponding to the postcode area and district for 108 farms.

Farm locations were indicated by the “outcode,” the first 4 letters of the postcode, which corresponds to the postcode area and district (Figure 1). Latitude and longitude were identified using the UK grid reference finder (https://gridreferencefinder.com/).

Climate data were obtained from the National Center for Environmental Information National Oceanic and Atmospheric Administration's Global Summary of the Day (NOAA National Centers for Environmental Information, 1999). Daily mean temperature, maximum temperature, and dewpoint temperature from 263 weather stations across the United Kingdom between longitudes 59.779 and 49.781 and latitudes 7.910 and 2.201 were obtained from January 1, 2006, to December 31, 2021. Of the stations obtained, 177 had data from each year, and stations were excluded if >10% of daily observations were missing in the year (1 station). Farms were matched to their nearest weather station based on distance from their point location (mean distance = 28.6 km, range = 4.3–66.9 km) using *geopandas* (Jordahl et al., 2020) in Python v 3.10.5.

A maximum thermal discomfort index (Thom, 1959) for each day was calculated as follows, using the maximum temperature-humidity index (**THI_max_**):

THI_max_ = 0.8 × T + [RH/100 × (T − 14.4)] + 46.4,

where T was the daily maximum temperature for the day and RH was the minimum relative humidity for the day.

The minimum RH for the day was calculated as follows:

RH = 100 × exp[17.625 × DP/(243.04 + DP)]/exp[17.625 × T/(243.04 + T)],

where DP is dewpoint temperature (°C) and T is the maximum temperature (°C) for the day.

**Figure 2:**
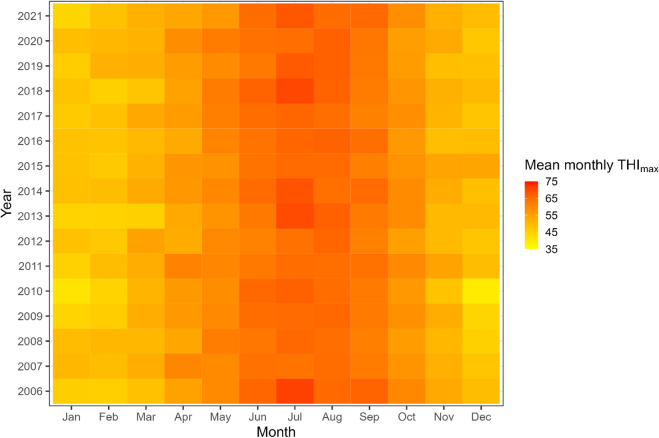
Summary of the mean monthly THI_max_ for 83 herds composing dataset 1. Data were obtained from 627,962 weather observations between 2006 and 2021.

Mean THI_max_, summarized for each month and year, is presented in Figure 2.

#### Dataset 2, Langhill Research Herd: Health Events and Treatment Records

The Langhill research herd are regularly mobility scored on a scale of 1 to 5 (Manson and Leaver, 1988), and body condition scored on a scale of 0 to 5 from the National Institute for Research in Dairying (Mulvany, 1977) and have detailed health records for all health events and medicine use. The health records included for the Langhill herd in the current dataset were as follows:

1. Health events: the date the cow was recorded as having a significant health event (see Supplemental Table S2 [see Notes] for a comprehensive list and frequency of health events recorded),
2. BCS: the dates and scores when the cow's BCS was recorded,
3. Mobility scores: the dates and scores when the cow's mobility score was recorded, and
4. Treatment: the dates and products used to treat illness, which were then filtered for whether the use category was anti-inflammatory or antibiotic.

#### Climate Records and Calculation of a Thermal Discomfort Index

**Figure 3:**
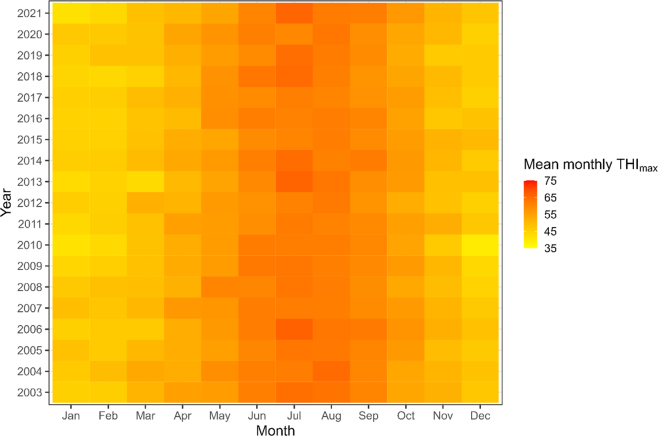
Summary of the mean monthly THI_max_ for the Langhill research herd. Data were obtained from 6,809 weather observations between 2003 and 2021.

From the National Center for Environmental Information National Oceanic and Atmospheric Administration's Global Summary of the Day (National Centers for Environmental Information, 2022) database, climate data were obtained from the Dundrennan weather station (∼38 km from the Langhill herd). The THI_max_ for each date (Figure 3) was calculated as above. One year (2004) was missing >10% of data, with only 319 of 366 d with daily observations, but is still presented and included in the subsequent analysis.

#### Datasets 1 and 2: Windows of Events During Pregnancy and Potential for Developmental Programming

For each pregnancy, an estimated date of conception was used for dataset 1 (283 d before the calving date) and the date of a cow's last insemination before pregnancy was considered the conception date for the Langhill cows. We also investigated 7 d before the estimated conception date because the preconception uterine environment can have lasting effects on health status of the offspring (Berry et al., 2008; Stephenson et al., 2018). Stressor events can have different effects on the fetus at different times during pregnancy and so several “windows” for events were considered, including the following:

• Trimester 1: 7 d preconception to 94 d of pregnancy—During T1, early embryonic development takes place. The body plans are established, with the majority of the organs having started to develop by d 40 (Winters et al., 1942) and the fetus begins to increase in size (Eley et al., 1978).
• Trimester 2: 95 to 189 d of pregnancy—During T2, the fetus continues to grow (Reynolds et al., 1990) and structures begin to be established, such as the number of myocytes in muscle fibers (Du et al., 2010).
• Trimester 3: 190 to 283 d of pregnancy—During T3, the majority of increase in fetal tissue size takes place (Winters et al., 1942), as well as proliferation of immune cells (Higgins et al., 1983), adipogenesis (Fève, 2005), and muscular development, including myocyte size and intramuscular adipocyte formation (Du et al., 2010).

Within each trimester window, the following were summarized in both datasets:

1. Presence or absence of each health event for each dam;
2. Mean THI_max_: mean value of all the daily values of THI_max_ between the relevant dates.

For dataset 1, because the majority of farms had monthly milk recordings, we considered the following milk quality variables within each trimester window:

1. Fat: minimum, median, and maximum percentage. This was categorized into >0%–3%, >3%–5%, >5%, and missing (if there was no recording between the trimester window dates).
2. Protein: minimum, median, and maximum percentage. This was categorized into >0%–3%, >3%–4%, >4%, and missing.
3. Fat/protein ratio:maximum ratio. This was categorized into >0–1, >1–1.2, >1.2–1.4, >1.4, and missing.
4. SCC: maximum SCC (100,000 cells). This was categorized into >0–50, >50–100, >100–200, >200–400, >400, and missing.
5. Milk yield: minimum, median, and maximum (L). This was categorized into >0–20, >20–30, >30–40, >40, and missing.

For the Langhill herd, we also considered:

1. Average BCS: under (<1.5), normal (1.5–3.25), and over (>3.25)
2. Locomotion score (**LS**): lame (LS_max_ ≥4), not lame (LS_max_ <4); see the “Dataset 2, Langhill Research Herd: Health Events and Treatment Records” section for details of the scoring system.

In the Langhill herd, recording of BCS and LS begins when the cow first enters the herd, after giving birth to her first calf. Because of this, data for parity 1 cows was not available and therefore not included in the final model.

Shorter intervals of pregnancy were considered, but there were insufficient data per window for the health events to allow analysis, particularly in dataset 1.

### Cow-Level Features

Features that were relevant to each calf were also included in the models; these were as follows:

1. Their mother's LRS, to provide a proxy for possible genetic effects because traits that make up the LRS (e.g., milk yield) are heritable. This predictor was centered around the mean mother LRS for the entire dataset.
2. Season of birth: calf season of birth, based on date of birth, was included to account for any potential confounding influence of birth season (spring = March–May, summer = June–August, autumn = September–November, and winter = December–February).

For the Langhill herd, a fixed effect was tested for the genetic group and the feed trial a cow was in.

### Farm-Level Features

For dataset 1, where multiple herds were considered, farm-level features were included to determine whether they affected the LRS of calves born on that farm. These were as follows:

1. Mean 305-d yield: for each calf, the mean 305-d yield of the herd at the time of the calf's birth was calculated as the mean of all the 305-d yields from all lactations that had occurred before the day of birth of the calf in the past 12 mo from the selected subset of cows.
2. Mean parity structure: a yearly mean parity structure for each farm was calculated as a proxy for the expected survival of a cow. This was calculated as the mean of the parity of mothers on the farm in the year of birth of the calf, including those that were born before 2006.
3. Farm: farm was included as a random effect to account for other unknown farm factors that differed among farms, such as diet and housing.

### Associations Between Explanatory Variables

Correlations between explanatory variables were tested by calculation of the Spearman's rank correlation coefficient, using the *stats* package in R (R Core Team, 2022).

### Modeling Associations Between Explanatory Variables and Lifetime Resilience Score

For dataset 1, linear mixed effects models using the *lmer* package (Bates et al., 2015) in R v4.2.2 (R Core Team, 2022) were used to identify whether events that occurred while the calf was in utero were associated with the lifetime resilience score of that calf.

The models took the following form:

*y_ijk_* = *β*_0_ + *β*_1_**x***_ijk_* + *β*_2_**x***_jk_ + β*_3_**x***_k_* + *f_k_* + *u_jk_* + *e_ijk_*,

where *y_ijk_* is the continuous outcome variable lifetime resilience score for calf *i* from dam *j* in herd *k*, *β*_0_ is the model intercept, **x***_ijk_* is the matrix of the explanatory variables at calf level and *β*_1_ their coefficients, **x***_jk_* is the matrix of the explanatory variables at dam level and *β*_2_ their coefficients, and **x***_k_* is the matrix of the explanatory variables at the farm level and *β*_3_ their coefficients. Residual error variance estimates were included at farm (*f_k_*), dam (*u_jk_*) and calf (*e_ijk_*) level and assumed to be normally distributed with mean = 0 and variances σ*_f_*, σ*_u_*, and σ*_e_*, respectively. Models were fitted using maximum likelihood.

Models were built using a forward stepwise selection process, adding variables where *P* < 0.05 (Wald's test of significance). Milk quality and yield variables were grouped into subgroups consisting of the minimum, median, and maximum for each variable, and if multiple were significant, the one with the lowest *P*-value was retained in the model and correlations between variables noted.

Polynomial terms (up to third degree) were tested in the final model for all continuous predictors. Interactions between biologically plausible variables were tested and were included if they were significant and improved model fit. Model fit was assessed by using calculation of the marginal and conditional R^2^ for mixed effects models (Nakagawa et al., 2017) and by leave-one-out cross-validation (**LOOCV**), training the model on all but one farm, and predicting values for the omitted farm.

A further set of analyses was conducted to evaluate possible associations between potential stress events during pregnancy and lifetime resilience score of granddaughters. That is, the outcome variable was the granddaughters LRS and the explanatory variables related to events during the pregnancy of the grandmother. The LRS of both the mother and grandmother were tested in the models as explanatory variables. The dataset comprised 1,586 granddaughters that could be matched to pregnancies of the original dams in the dataset, from 65 farms, and analyses were conducted as described above.

For dataset 2, the models took the form

*y_ij_* = *β*_0_ + *β*_1_**x***_ij_* + *β*_2_**x***_j_* + *u_j_* + *e_ij_*,

where *y_ij_* is the continuous outcome variable lifetime resilience score for calf *i* in from dam *j*, *β*_0_ is the model intercept, **x***_ij_* is the matrix of the explanatory variables at calf level and *β*_1_ their coefficients, and **x***_j_* is the matrix of the explanatory variables at dam level and *β*_2_ their coefficients. Residual error variance estimates were included at dam (*u_j_*) and cow (*e_ij_*) level and assumed to be normally distributed with mean = 0 and variances *σ_j_* and *σ_ij_*, respectively. Models were fitted using maximum likelihood.

The model fitting process was as described as above, with LOOCV validation performed by leaving out one genetic/feed trial “group” at a time.

## RESULTS

### Descriptive Statistics—LRS and Health Events

**Figure 4:**
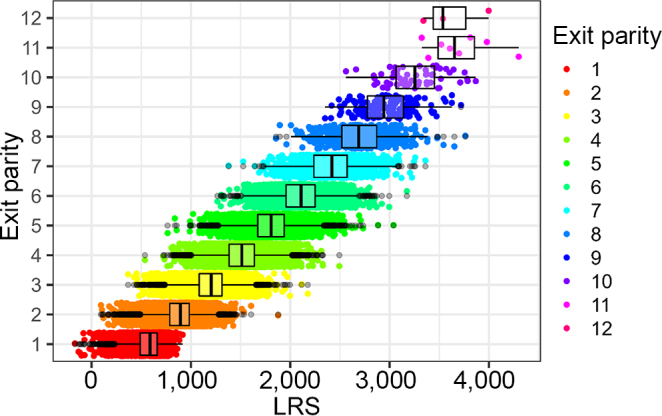
Distribution of LRS from 42,982 cows from 83 herds by exit parity (the parity at which the cow left the herd). Boxes show the median and 25th and 75th percentiles, while the whiskers extend from the hinge to the largest and smallest values no further than 1.5× the interquartile range. Data beyond the edge of the whiskers are plotted as individual points (shown in black).

**Figure 5:**
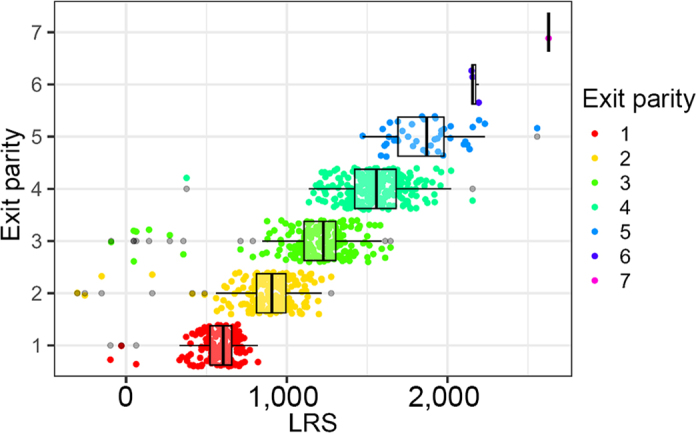
Distribution of LRS from 811 cows from the Langhill research herd by exit parity (the parity at which the cow left the herd). Boxes show the median and 25th and 75th percentiles, while the whiskers extend from the hinge to the largest and smallest values no further than 1.5× the interquartile range. Data beyond the edge of the whiskers are plotted as individual points (shown in black).

**Figure 6:**
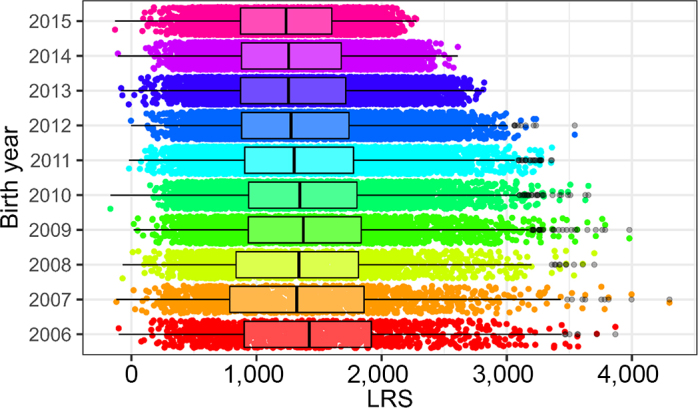
Lifetime resilience scores by year of birth for 42,982 cows from 83 herds (dataset 1) by year of birth. Boxes show the median and 25th and 75th percentiles, while the whiskers extend from the hinge to the largest and smallest values no further than 1.5× the interquartile range. Data beyond the edge of the whiskers are plotted as individual points (shown in black).

**Figure 7:**
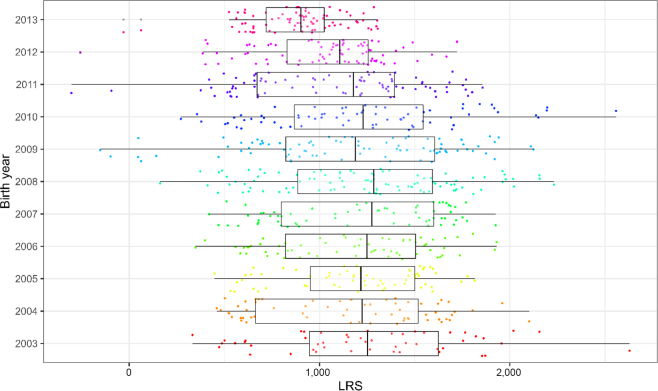
Lifetime resilience scores by year of birth for 811 cows from the Langhill research herd (dataset 2). Boxes show the median and 25th and 75th percentiles, while the whiskers extend from the hinge to the largest and smallest values no further than 1.5× the interquartile range. Data beyond the edge of the whiskers are plotted as individual points (shown in black).

We calculated 42,982 resilience scores from the 83 herds with sufficient recording data and 811 resilience scores for cows with sufficient data from the Langhill research herd. As expected, cows that had completed more lactations tended to have higher scores (dataset 1: Figure 4, Langhill: Figure 5). The LRS ranged from −168 to 4,300 in dataset 1 and from −303 to 2,629 in dataset 2 (Langhill), and resilience scores did not appear to increase over time (dataset 1: Figure 6; Langhill: Figure 7).

### Associations Between Events that Occurred while the Calf was In Utero and LRS of Daughters

In dataset 1, a health event (excluding use of dry cow therapies) occurred in a mean 22% of the pregnancies across the farms (Table 3), where cows were in either their second or subsequent pregnancies. Use of antimicrobial products was the most common health event (13% of pregnancies), followed by mastitis (11% of pregnancies). A summary of the milk quality variables over the pregnancies is in Supplemental Table S1 (see Notes) and correlations between all explanatory variables are in Supplemental Figure S1A–D (see Notes).

**Table 3:** Mean proportion of pregnancies with lameness, mastitis, and antibiotic and anti-inflammatory usage from dams of calves born from 2006 to 2015 in the recording years on 83 farms^1^

Stressor	Mean	Median	Minimum	Maximum
Anti-inflammatory use	0.02	0.00	0	0.42
Antimicrobial use (excluding dry cow therapies)	0.13	0.00	0	0.75
Antimicrobial use (including dry cow therapies)	0.32	0.00	0	1.00
Lameness	0.02	0.00	0	0.37
Mastitis	0.11	0.09	0	0.32
Any stressor (including dry cow therapies)	0.39	0.23	0	1.00
Any stressor (excluding dry cow therapies)	0.22	0.16	0	0.76

1 Data was obtained from 9,292 calves from 7,334 mothers that were in their second pregnancy or greater.

In the Langhill herd, health events occurred in a mean of 52% of pregnancies, with the largest proportion of health events being recorded in trimester 1 of pregnancies (35%; Table 4). Use of antimicrobial products was common (70% of pregnancies) with the use of anti-inflammatories low (0.02% of pregnancies). At some point during pregnancy 77% of cows were recorded as having a high LS (≥4), and just 0.07% of cows were recorded as having a low or high body condition score (<1.5, >3.5).

**Table 4:** Mean proportion of pregnancies with stressors recorded from dams of calves born from 2006 to 2013^1^

Stressor^2^	Mean_All_^3^	Mean_F25_	Mean_T1_	Mean_T2_	Mean_T3_
Anti-inflammatory use	0.02	0.00	0.01	0.01	0.02
Antibacterial use	0.70	0.13	0.41	0.33	0.19
High locomotion score	0.77	0.21	0.51	0.51	0.39
Low or high BCS	0.07	0.05	0.04	0.03	0.03
Health event	0.52	0.12	0.35	0.21	0.19

1 Table contains data from the animals that were included in the final model, 192 calves from 156 mothers.

2 High locomotion score was based on average scores recoded (≥4). Low or high body condition score was based on average scores recorded (<1.5, >3.5).

3 Mean_All_ = mean for entire pregnancy; mean_f25_ = mean for first 25 days of pregnancy; mean_T1_ = mean for trimester 1; mean_T2_ = mean for trimester 2; mean_T3_ = mean for trimester 3.

The final mixed effects model of calf LRS and in utero events in the mother for dataset 1 is presented in Table 5. A higher mean daily THI_max_ in the first and third trimester of pregnancy was associated with lower LRS. Calves that were born to older dams (dams in their third or higher pregnancy compared with dams in their second pregnancy) had lower LRS. Higher LRS for mothers were associated with higher LRS for their calves (Table 5).

**Table 5:** Final model of calf lifetime resilience scores and in utero events in the mother in dataset 1^1^

Predictor	N	β	LCI to UCI	*P*-value
Intercept	—	1,915.00	1,466.35 to 2,363.65	<0.001
Fixed effects				
Mean THI_max_, T1	9,292	−5.18	−9.21 to −1.16	0.012
Mean THI_max_, T3	9,292	−5.76	−9.81 to −1.71	0.005
Pregnancy 2	4,756	—		
Pregnancy 3	2,684	−38.40	−62.70 to −14.10	0.002
Pregnancy 4	1,852	−74.47	−104.26 to −44.68	<0.001
Mother LRS	9,292	0.07	0.05 to 0.09	<0.001
Minimum milk yield, T1				
>20–30 L	3,753	—		
>0–20 L	1,760	−54.65	−85.52 to −23.78	0.001
>30–40 L	2,754	1.45	−24.72 to 27.61	0.914
>40 L	715	−5.56	−50.37 to 39.25	0.808
Data missing	310	94.47	8.14 to 180.79	0.032
Maximum milk yield, T3				
>20–30 L	3,206	—		
>0–20 L	3,555	4.79	−21.35 to 30.93	0.720
>30–40 L	893	−4.73	−42.31 to 32.85	0.805
>40 L	118	−104.20	−196.86 to −11.53	0.028
Data missing	1,520	5.83	−27.55 to 39.22	0.732
Median milk fat, T1				
>3%–5%	6,593	—		
>0%–3%	844	−44.09	−81.34 to −6.84	0.020
>5%	823	14.79	−26.90 to 56.47	0.487
Data missing	1,032	−121.48	−186.21 to −56.75	<0.001
Random effects, SD				
Residual	—	477.17		
Dam	7,334	81.42		
Farm	83	142.27		

1 N = number of observations; β = model coefficient; LCI = lower confidence interval; UCI = upper confidence interval; *P*-value = *P*-value from Wald's test of significance; T = trimester.

Milk yield and quality variables over the mother's pregnancy were associated with daughter LRS. Daughter LRS were lower where milk yields were low in trimester 1 (>0–20 L compared with >20–30 L), where median fat percentages in trimester 1 were 0%–3% compared with >3–5%, and when milk yields were high (>40 L compared with >20–30 L) in trimester 3 (Table 5).

Overall, the model explained a low proportion of the variation in LRS (12%, conditional R^2^ = 0.120), with the fixed effects explaining 1% of this (marginal R^2^ = 0.0116). Plots of residuals versus fitted values (Supplemental Figure S2, see Notes) and predictions from the LOOCV cross-validation (Supplemental Figure S3A and S3B, see Notes) indicated a good model fit.

The final model of calf LRS and in utero events in the mother for dataset 2 (Langhill) is presented in Table 6. Calves that were born to older dams (dams in their fourth or greater pregnancy compared with their second pregnancy) had lower LRS. Calves whose mothers had a maximum LS of ≥4 in the third trimester of pregnancy had lower LRS than calves whose mothers had maximum locomotion scores less than <4 in the third trimester of pregnancy.

**Table 6:** Final model of calf lifetime resilience scores and in utero events in the mother, model coefficients and Wald's confidence intervals and *P*-values for the Langhill research herd (dataset 2)^1^

Predictor	N	β	LCI to UCI	*P*-value
Intercept	—	1292.52	1209.11 to 1375.93	<0.001
Fixed effect	—	—	—	—
Pregnancy 2	102	—	—	—
Pregnancy 3	60	−22.40	−137.36 to 92.56	0.701
Pregnancy 4+	30	−178.93	−329.75 to −28.12	0.020
Maximum locomotion score, T3				
<4	118	—		
>4	74	−151.12	−260.50 to −41.75	0.007
Random effect, SD	—	—	—	—
Residual	—	324.77	—	—
Dam	155	185.67	—	

1 N = number of observations; β = model coefficient; LCI = lower confidence interval; UCI = upper confidence interval; *P*-value = *P*-value from Wald's test of significance; T = trimester; SD = standard deviation.

Overall, the model explained a low proportion of the variation in LRS (∼30%, conditional R^2^ = 0.298), with the fixed effects explaining about 7% (marginal R^2^ = 0.069). Plots of residuals versus fitted values (Supplemental Figure S4, see Notes), and predictions from the LOOCV cross-validation (Supplemental Figure S5A and S5B, see Notes) indicated a good model fit.

### Associations Between Events that Occurred while the Mother Was In Utero and Lifetime Resilience of Granddaughters

The final model for granddaughters in dataset 1 is presented in Table 7. Granddaughters had lower LRS when their grandmother was in their third pregnancy compared with their second and when their grandmother had received an antimicrobial treatment during trimester 3. Granddaughters had higher resilience scores when SCC_max_ counts were 201,000 to 400,000 in trimester 2 compared with 0 to 50,000.

**Table 7:** Final model of calf lifetime resilience scores and in utero events in the grandmother (65 farms, 1,586 granddaughters)^1^

Predictor	N	β	LCI to UCI	*P*-value
Intercept		1197.96	1138.63 to 1257.29	<0.001
Fixed effects				
Pregnancy 2	987			
Pregnancy 3	409	−57.37	−108.88 to −5.87	0.029
Pregnancy 4+	190	−53.32	−125.53 to 18.89	0.148
Antimocrobial use, T3				
No	1502	—		
Yes	84	−106.29	−197.42 to −15.16	0.022
Maximum SCC, T2				
0–50	362	—		
51–100	375	56.22	−7.87 to 120.31	0.086
101–200	416	29.08	−34.02 to 92.18	0.366
201–400	221	87.88	12.52 to 163.25	0.022
>400	172	46.12	−37.91 to 130.14	0.282
Data missing	40	−101.06	−246.35 to 44.22	0.173
Random effects, SD				
Residual	—	420.05		
Cow	1227	94.38		
Farm	65	126.85		

1 N = number of observations; β = model coefficient; LCI = lower confidence interval; UCI = upper confidence interval; *P*-value = *P*-value from Wald's test of significance; T = trimester, and SD = standard deviation. The SCC value is given as ×1,000.

Plots of residuals versus fitted values and predictions from LOOCV validation indicated good model fit (Supplemental Figures S6, S7A, S7B; see Notes); however, the fixed effects explained only a very small proportion of variation in LRS (∼1%, marginal R^2^ = 0.014, conditional R^2^ = 0.136). In the Langhill herd, no in utero event predictor variables were significantly associated with LRS of granddaughters, but this dataset was very small (74 granddaughters).

## DISCUSSION

This is the first study to explore associations between LRS of dairy cows and events that occurred in utero in a large longitudinal dataset of dairy cattle. The importance of early life events in determining future performance of dairy cattle is becoming increasingly apparent and the key findings from our study were that cows that experienced higher THI_max_ values in the first or last trimester of pregnancy, cows that were born to multiparous dams compared with primiparous dams, calves from cows with the lowest milk yields and fat percentages in the first trimester, calves from cows with high milk yields in the third trimester, and those born to dams with high locomotion scores in the third trimester had lower LRS. This adds to the existing evidence base that the in utero environment has lifelong implications on calf performance.

Currently, relatively little is known about the exact mechanisms of developmental programming events, but they tend to result in either alterations to tissue and organ structures or functional alterations that arise from changes in gene expression (Reynolds and Caton, 2012). In laboratory animals, some specific links between maternal environment and offspring performance have been reported; for example, in rats, maternal malnutrition is associated with the occurrence of prostatic disorders in the offspring (Portela et al., 2021) and in mice, depriving the mother of water during pregnancy is associated with dysregulation of plasma glucose levels and fatty liver in female offspring (Kondo et al., 2023). In our study, we have identified several potential effects, but additional research is required to elucidate underpinning mechanisms.

The effects of fetal heat stress in dairy cattle have been reported mostly in late gestation, and the results of our study are consistent with this (Table 5). Calves born to late-gestation heat-stressed dams weighed less both at birth and up to one year of age (Collier et al., 1982; Monteiro et al., 2016; Laporta et al., 2017; Dado-Senn et al., 2020), have compromised metabolic and immune functions (Dado-Senn et al., 2020), and have poorer milk yield and shorter life spans (Monteiro et al., 2016; Laporta et al., 2020; Skibiel et al., 2018b; Weller et al., 2021). All of these factors potentially lead to lower LRS. Heat stress may be particularly detrimental in late gestation, when the majority of increase in fetal tissue size takes place (Winters et al., 1942). Additionally, the effects of heat stress on the mother can lead to behavioral and physiological changes that contribute to dysregulation in fetal growth by reducing the nutrition available to the fetus as nutrition is associated with growth (Funston et al., 2010). For example, increased maternal core body temperature leads to a reduction in DMI (Lamp et al., 2015) and a redirection of blood from the gravid uterus to the periphery to limit the increase in temperature to the fetus (Reynolds et al., 1990).

We also identified that calves that experienced higher mean THI_max_ values in early gestation had lower LRS (Table 5). Further investigation is needed to determine exactly how heat stress in early gestation is associated with lifetime performance of cattle, but embryos are known to be sensitive to heat stress in the early stages of pregnancy. Changes that have been associated with heat stress during embryo development include changes in DNA methylation (Paula-Lopes and Hansen, 2002) and increased production of reactive oxygen species, leading to cellular damage (de Barros and Paula-Lopes, 2018). Many embryos do not survive early heat stress exposure in cattle, leading to pregnancy loss (García-Ispierto et al., 2006; Sakatani et al., 2008); however, in this study we were unable to assess any effect on early embryonic loss. We did not find any effect of THI_max_ in the Langhill herd; however, this herd is housed in Scotland where the values of daily THI_max_ experienced did not reach what could be considered heat stress (Figure 3). The physiological effects of heat stress, such as decline in milk production are seen at THI values of ≥68 (Morton et al., 2007; Gantner et al., 2017). Most of the herds in dataset 1 were in Southern England (Figure 1) and herds in the south are more likely to experience temperatures that could lead to heat stress (Dunn et al., 2014). Due to the nature of the data available, there were limitations in the assessment of heat stress because farm-specific information was not available and data from local weather stations were used. We acknowledge that these measurements are limited as they were not able to take into account factors such as air flow or availability of shade, ventilation, and cooling equipment or factors such as photoperiod that may differ between farms or even between animals on the farm. Therefore, animals may not have experienced the exact THI_max_ as measured, yet despite this source of random error, clear relationships were still identified in the final models.

The lower performance phenotype of calves that had experienced higher THI_max_ is likely because heat stress is known to affect several of the components that make up the LRS. Age at first calving is lower for heifers born to mothers that were not cooled during pregnancy (Dahl et al., 2016) and these animals also produce less milk as heifers (Monteiro et al., 2016; Skibiel et al., 2018b). Lower milk yields likely result from the fact that heat stress while in utero is associated with smaller alveoli and greater proportions of connective tissue in the mammary gland (Skibiel et al., 2018b). Many differentially methylated genes involved in processes such as cellular repair, oxidative defense, and energy metabolism are found in calves that have experienced fetal heat stress (Skibiel et al., 2018a) and resulting epigenetic changes may contribute to the lower LRS seen for calves from mothers who had experienced higher THI_max_, although this is still an emerging area of research. Another explanation is body weight; in utero heat-stressed calves are lighter (Tao et al., 2012; Dahl et al., 2016; Monteiro et al., 2016) and because heavier heifers reach puberty faster (Archbold et al., 2012) and age at first calving is a component of the LRS, body weight may partially explain the poorer performance of these calves.

Calves born to mothers of a higher parity and therefore older animals had lower LRS (Table 5, Table 6) and this effect was also seen for granddaughters in dataset 1 (Table 7). This aligns with a previous study that reported that the highest yielding daughters in a cohort were born to younger mothers (Astiz et al., 2014). In the current study, it was not possible to discriminate whether the effect of parity was due to the cow having had previous pregnancies or due to increased maternal age and possible epigenetic changes associated with aging. There are epigenetic effects associated with aging in cattle such as changes in DNA methylation (Ribeiro et al., 2022), but currently little is known about effects on the in utero environment caused by epigenetic changes; our results suggest this area is worthy of future research in terms of its effect on lifetime resilience.

The mother's LRS was included in the models as a fixed effect on the basis that the traits that make up the LRS, particularly milk yield, are heritable (Hill et al., 1983; Visscher and Goddard, 1995; Gudex et al., 2014), and therefore the mother's LRS would act as a proxy for genetic merit of the dam. Higher mother LRS was associated with higher LRS of the calf, suggesting a genetic component in resilience. There are genetic correlations between resilience indicators and health, fertility, and longevity (Twomey et al., 2018; Poppe et al., 2020, 2021), although resilience is a composite trait not currently incorporated into breeding programs (Berghof et al., 2019). In calculating the LRS for the Langhill dataset, the herd was split into “subherds” based on feed trial and genetic merit, with averages for each subherd contributing to the LRS equation. This was to take into account any effect feed-trial type or genetic merit may have had on LRS. Had the LRS of the Langhill herd been calculated without the categorization of the herd into subherds, an effect of mother LRS may have also been found in this dataset. We did not have sufficient data to examine the effect of sire on LRS although this would be of interest. There is evidence of sire effects on factors such as gestation length (Fang et al., 2019), which can be associated with performance of offspring because increased gestation lengths are associated with greater incidences of stillbirth, retained placenta, and metritis (Vieira-Neto et al., 2017). Sire effects could also affect resilience through genetic links between factors such as milk yield, age at first calving, or susceptibility to foot lesions (Oikonomou et al., 2013; Konkruea et al., 2017).

In our study, there was no effect of health events in the mother on calf LRS in dataset 1 (Table 5); however, a significant reduction in LRS occurred when mothers were lame in trimester 3 in the Langhill herd (Table 6). Lame cattle spend less time feeding and take in less feed (Miguel-Pacheco et al., 2014; Thorup et al., 2016) and because nutrition is associated with fetal tissue growth (Funston et al., 2010), this may cause alterations that lead to a reduced LRS for these offspring. Lameness in the Langhill herd was assessed using a 5-point scale, with cows considered to have a lameness event if a max mobility score of 4 or greater was recorded within a window-of-events period during pregnancy. There is more uncertainty about lameness records in the large dataset because farmers differ in what they recognize as lameness or determine as sufficient lameness to require treatment (Horseman et al., 2014).

Other studies have found links between clinical disease and performance of daughters (Carvalho et al., 2020). One limitation of dataset 1 was that we were not able to take into account the duration, frequency, or severity of health events due to the inconsistences of records between farms; some treatment events included details of the treatment used such as drug dose and length but others did not. Additionally, some events may have been missed because of recording errors, leading to misclassification of cows. However, we were able to look at this in more detail with the Langhill research herd, where events were known to be recorded with a high level of consistency and accuracy, which overall resulted in a higher proportion of these events occurring. The only clinical disease associated with LRS of calves was lameness, as discussed previously.

Milk quality factors were associated with lifetime performance. Milk quality variables were tested in the models for 2 reasons, first milk production is a major component of dairy cow energy balance and higher producing cows tend to be in greater negative energy balance (Berry et al., 2006), and second, perturbations in yield or quality could be indicative of disease or other metabolic or physiological disturbances that may not have been seen or recorded (Poppe et al., 2020; Kok et al., 2021). Our models revealed that the cows with low milk yields and fat percentages in trimester 1 (Table 5) had calves with lower LRS, which could be because low yield and fat percentage is indicative of either increased metabolic stress or unrecorded or unseen health issues, which may have subsequent deleterious effects on progeny performance. Other studies have reported associations between yields of dams and yields of their offspring, as well as composition (Berry et al., 2008) where higher milk fat concentration was associated with greater milk yield, reduced survival, and reduced somatic cell counts in the offspring. Using milk quality variables in our models meant we were unable to assess the LRS of the calves from mothers in their first parity as they did not have milk quality information available.

Our analysis of the LRS of granddaughters identified that LRS were lower in granddaughters of cows in their third pregnancy compared with their second and that received an antimicrobial treatment in trimester 3. Currently, there is limited understanding of carryover effects of maternal exposures on subsequent generations, although recent studies have reported an association between late-gestation heat stress in the grandmother and reduced milk yield and survival of the F2 progeny (granddaughters) to first lactation (Laporta et al., 2020). Again, this area warrants further research. We did not find any effects for granddaughters in the Langhill herd, which may be because the dataset is much smaller (74 cows).

A limitation of our final models is that they explained a relatively small percentage of the total variation in LRS (∼1% explained by the fixed effects in dataset 1 and 3% in dataset 2). This is unsurprising because many events that happen to a calf after birth will affect lifetime performance, and we did not include these aspects in the analyses. In dataset 1, there was more variation in LRS between farms than between dams (Table 5). The random effect for farm was included to account for factors that differ between farms but cannot be measured directly, such as housing or diet. Other studies using the same resilience scoring system have also found that LRS are difficult to predict across different farms (Adriaens et al., 2020), suggesting that the unidentified farm factors are important in determining calf LRS, which is unsurprising. It is also possible that policies within each farm changed over time, for example, changes in diet, housing, or culling policies, all of which could contribute to changes in within-farm LRS, although most components of the score were measured relative to the herd average, and therefore the one that would have the biggest effect would be a change in culling policy. However, we demonstrate that the in utero environment has a lasting effect on calf lifetime performance, and these factors warrant further research, particularly in the context of the challenges such as climate change that are facing the dairy industry.

## CONCLUSIONS

In conclusion, this research has demonstrated associations between events that occur during pregnancy and LRS in dairy cows. An increased THI during the first and the final trimester of pregnancy was associated with lower LRS and this may become of increasing importance in the face of global climate change.
